# Early epigenetic alterations in cardiac tissue following perinatal sertraline exposure in mice

**DOI:** 10.3389/fcvm.2026.1894882

**Published:** 2026-10-28

**Authors:** Yongjun Lu, Michael S. Chimenti, Sarah E. Haskell

**Affiliations:** 1 Stead Family Department of Pediatrics, University of Iowa Carver College of Medicine, Iowa City, IA, United States; 2 Iowa Institute of Human Genetics, University of Iowa Carver College of Medicine, Iowa City, IA, United States

**Keywords:** DNA methylation, mouse model, neonatal heart development, perinatal sertraline exposure, selective serotonin reuptake inhibitors, serotonin signaling, targeted bisulfite sequencing

## Abstract

**Introduction:**

Maternal use of selective serotonin reuptake inhibitors (SSRIs), including sertraline, has been associated with an increased risk of congenital heart defects, but the underlying mechanisms remain unclear. SSRIs cross the placenta and may influence cardiac development during sensitive periods. Previous work showed that perinatal sertraline exposure impairs postnatal growth, alters cardiomyocyte calcium handling, dysregulates serotonin signaling genes (*Htr2a*, *Htr2b*, and *Slc6a4*), and chronically reduces left ventricular size. We hypothesized that these effects may involve early epigenetic alterations in cardiac DNA methylation.

**Materials & methods:**

Adult female C57BL/6J mice received daily intraperitoneal injections of sertraline (5 mg/kg) or saline for five days before mating and throughout gestation. Offspring continued to receive 1.5 mg/kg of sertraline until postnatal day 14 (PN14), when hearts were collected. Global DNA methylation (5-methylcytosine, 5-mC) was quantified using a colorimetric ELISA assay. Targeted bisulfite sequencing (TBS-seq) profiled CpG methylation across predefined promoter and regulatory regions of 10 genes involved in serotonin signaling and cardiac function. Regional differential methylation was analyzed across 23 regions using overdispersion-corrected logistic regression with Benjamini–Hochberg (BH) adjustment.

**Results:**

Global 5-mC levels did not differ significantly between sertraline-exposed and control groups. TBS-seq identified 1,941 CpG sites across 25 targeted regions. After coverage and regional quality filtering, 1,411 CpG sites across 23 regions were retained. Regional analysis identified two regions with significant differential methylation after FDR correction: the *Htr2a* enhancer showed an 8.19% increase in methylation (*p* < 4.1 × 10^−15^, *q* < 9.4 × 10^−14^), whereas the *Tph2-*associated region showed a 3.00% decrease in sertraline-exposed animals (*p* < 0.0020, *q* < 0.0229). Secondary site-level analyses identified no individual CpG sites that remained significant after multiple-testing correction. Overall methylation profiles were highly correlated across samples, with no significant global difference between treatment groups.

**Discussion:**

Perinatal sertraline exposure was associated with localized regional DNA methylation changes in neonatal cardiac tissue without a global change in 5-mC. The *Htr2a* enhancer and *Tph2-*associated region represent candidate regulatory loci linking early-life SSRI exposure with cardiac serotonergic pathways. These findings support further investigation of regional epigenetic alterations using integrated methylation, transcriptomic, and cell-type-resolved approaches to determine their functional significance and potential contribution to later-life cardiovascular outcomes.

## 1 Introduction

Selective serotonin reuptake inhibitors (SSRIs) are prescribed to approximately 10%–15% of pregnant women in the United States to manage depression and anxiety disorders (1–5). Although generally considered safe for use during pregnancy, growing evidence suggests that prenatal SSRI exposure may exert subtle but lasting effects on fetal development beyond the central nervous system, and may increase the risk of congenital defects (6–10). Of particular concern is the developing cardiovascular system, where early disruptions may contribute to long-term structural and functional alterations (11–17).

Serotonin (5-hydroxytryptamine, 5-HT) plays a critical role in cardiac development by regulating key cellular processes such as proliferation, migration, and differentiation during embryogenesis (18). SSRIs, including sertraline, inhibit the serotonin transporter (*Slc6a4/*SERT), leading to increased extracellular 5-HT levels (18). As SSRIs readily cross the placenta, the fetus may be exposed to elevated serotonin levels during critical windows of heart development. Animal models have shown that modulation of SERT activity during development can lead to cardiac pathologies, including fibrosis, valvulopathy, and cardiomyopathy (19, 20).

While the incidence of major congenital heart defects associated with SSRI use is relatively low, several clinical and preclinical studies, including our own, have reported altered cardiac phenotypes following perinatal SSRI exposure (21–25). We previously demonstrated that sertraline treatment impaired postnatal growth, disrupted calcium oscillations in cardiomyocytes, upregulated specific miRNAs together with downregulation of serotonin signaling components such as *Htr2a*, *Htr2b*, and *Slc6a4,* and chronically reduced left ventricular size (25). These findings raise the possibility that SSRI exposure interferes with gene regulatory programs during heart development.

One potential mechanism underlying these persistent changes is epigenetic modification, particularly DNA methylation. DNA methylation is crucial for regulating gene expression, influencing chromatin structure, transcription factor activity, and other regulatory pathways important in development, cellular differentiation, and maintaining tissue-specific gene expression patterns (26, 27). Methylation at CpG dinucleotides within promoter or enhancer regions is especially critical during organogenesis (28–32). Environmental insults during sensitive developmental periods may alter DNA methylation patterns, potentially contributing to changes in gene expression and organ function that can persist into adulthood (33–35). Recent studies also underscore the importance of the gestational environment and prenatal stress on epigenetic programming, with implications for lifelong health outcomes (36).

In this study, we investigated whether perinatal sertraline exposure is associated with locus-specific DNA methylation differences in neonatal cardiac tissue. We employed targeted bisulfite sequencing (TBS-seq) to examine DNA methylation at CpG sites within promoter and regulatory regions of genes implicated in serotonin signaling, cardiac contractility, and calcium handling. Additionally, we quantified global 5-methylcytosine (5-mC) levels using a colorimetric ELISA assay to assess broader methylation changes. Our aim was to identify gene-specific DNA methylation differences that may represent early epigenetic markers and candidate loci for future mechanistic investigation following developmental SSRI exposure.

## 2 Methods & materials

### 2.1 Animals & sertraline exposure model

All experimental procedures were conducted in accordance with the Animal Welfare Act and the National Institutes of Health Guide for the Care and Use of Laboratory Animals. Protocols were approved by the Institutional Animal Care and Use Committee (IACUC) at the University of Iowa.

Adult female C57BL/6J mice (−10 weeks old) were obtained from Jackson Laboratory (Bar Harbor, ME) and used to generate sertraline-exposed offspring, following previously established protocols (24, 25). C57BL/6J mice, an isogenic strain, were used to minimize genetic heterogeneity, a known confounder in DNA methylation analyses (37). Prior to mating, females received daily intraperitoneal (IP) injections of either saline (0.9% sodium chloride Injection, USP; Hospira, Lake Forest, IL) at 10 mL/kg or sertraline (5 mg/kg; Cat# S6319, Sigma-Aldrich, St. Louis, MO) for five consecutive days. Treatment continued throughout pregnancy until delivery.

To maintain perinatal sertraline exposure across a developmental window corresponding approximately to late gestation (third trimester) in humans, maternal sertraline treatment was administered throughout pregnancy, followed by daily intraperitoneal injections of sertraline (1.5 mg/kg) or saline (3 mL/kg) directly to offspring from postnatal day 0 (PN0) through PN14. This exposure paradigm therefore represents combined gestational and early postnatal exposure. The PN0–PN14 developmental period in mice coincides with active cardiomyocyte proliferation and is considered developmentally analogous to the third trimester of human gestation (38, 39). Following euthanasia, neonatal left ventricles were collected for analysis.

For targeted bisulfite sequencing, litters from each treatment group were randomly selected, and one pup from each selected litter was used for cardiac tissue collection and DNA methylation analysis (sertraline, *n* = 4; saline, *n* = 4). Each biological replicate therefore represented a different litter, with two males and two females included in each treatment group. Sample-level metadata, including treatment group, dam/Litter ID, sex, assay, and sequencing batch, are provided in Supplementary Table S2.

### 2.2 Total DNA extraction and purification

Approximately 25 mg of left ventricular tissue was minced into small fragments and placed into a 1.5 mL microcentrifuge tube containing lysis buffer. Tissue homogenization was performed using Pellet Pestles (Sigma-Aldrich, Cat# Z359971 and Z359963). To ensure RNA-free genomic DNA, RNase A (10 mg/mL; Thermo Scientific, Cat# EN0531) was added to the lysate.

Total DNA extraction and purification were carried out using the spin-column protocol provided in the DNeasy Blood & Tissue Kit (Qiagen, Cat# 69504). DNA concentrations were quantified using the Qubit™ dsDNA High Sensitivity (HS) Assay Kit (Invitrogen, Cat# Q32851) with a Qubit 4 Fluorometer (Thermo Fisher Scientific).

### 2.3 Global 5-methylcytosine (5-mC) quantification

A colorimetric enzyme-linked immunosorbent assay (ELISA) was employed to assess global DNA methylation (5-mC) levels. This method relies on specific antigen–antibody interactions that trigger enzymatic reactions, resulting in a color change in the reaction medium that can be quantified spectrophotometrically (40).

In this study, 5-mC levels in cardiac tissue were assessed using a colorimetric ELISA-based assay (Global DNA Methylation Assay Kit; Abcam, Cat# ab233486) according to the manufacturer's instructions. Genomic DNA was immobilized onto strip wells pretreated to enhance DNA binding. Methylated cytosines were recognized by a 5-mC-specific capture antibody and subsequently detected using a proprietary detection system provided with the kit. Colorimetric quantification was performed by measuring absorbance at 450 nm using a microplate reader (Molecular Devices, SpectraMax M2).

The percentage of 5-mC in total DNA was calculated using the following equation:

$$5mC\text{\%}=\frac{Sample\;OD-Negative\;control\;OD}{4\times slope\times dG}$$

Where: OD is the optical density at 450 nm, slope is determined by linear regression from standard curve data, dG is the amount of guanine in the sample, assumed to be 25% of the total DNA mass, based on the equal distribution of nucleotides (A, T, G, C). Guanine content is used as a proxy for cytosine, assuming equivalence in unmethylated DNA.

While this assay provides an overall measure of global 5-mC levels, it does not resolve methylation at individual CpG sites. Therefore, gene-specific methylation analyses remain essential for detailed epigenetic investigations.

### 2.4 Region-specific DNA methylation analysis by targeted bisulfite sequencing (TBS-seq)

Four litters from each treatment group were randomly selected, and one pup from each selected litter was used for cardiac tissue collection and DNA methylation analysis (sertraline, *n* = 4; saline, *n* = 4). Thus, each biological replicate represented a different litter, and multiple pups from the same litter were not included in the sequencing analysis.

TBS-seq is a high-resolution, capture-based approach for region-specific DNA methylation analysis. In the present study, TBS-seq was performed using the xGen™ Methyl-Seq DNA Library Prep Kit with hybridization capture (Integrated DNA Technologies, Coralville, IA, USA) (41). Similar approaches have been reported previously for capture-based bisulfite sequencing methods (27, 42–46). By combining DNA fragmentation, bisulfite conversion, and targeted capture with next-generation sequencing, TBS-seq enables quantitative measurement of methylation at individual CpG sites within predefined genomic regions. TBS-seq was selected because the objective of this study was to obtain high-depth, quantitative DNA methylation profiles of predefined candidate loci associated with serotonergic signaling, cardiac calcium handling, and cardiac development, rather than to perform an unbiased genome-wide methylome analysis. By concentrating sequencing depth within selected genomic regions, TBS-seq enables focused characterization of methylation at predefined candidate loci within a hypothesis-driven study design. Whole-genome bisulfite sequencing (WGBS) provides broader genome-wide coverage but was not required for the targeted objectives of the present study. Reduced representation bisulfite sequencing (RRBS), in contrast, enriches genomic regions according to restriction-enzyme sites and therefore does not provide the same direct control over selection of the predefined candidate regions examined here (45, 46).

#### 2.4.1 Mechanical shearing of genomic DNA fragments

Fragmentation of high-molecular-weight genomic DNA is a critical preparatory step for next-generation sequencing. Genomic DNA extracted from postnatal day 14 (PN14) mouse heart tissue was resuspended in low-EDTA TE buffer (10 mM Tris-HCl, 0.5 mM EDTA, pH 8.0) and transferred into Covaris microTUBE AFA® Fiber Snap-Cap tubes (Covaris, Cat# 520045). Mechanical shearing was performed using the Covaris E220 Focused-ultrasonicator under manufacturer-recommended conditions to produce DNA fragments of desired size (centered around 300–500 bp for downstream application). The volumes of the DNA samples was adjusted and held constant at 130 μL with a concentration of 50 ng/μL to obtain the same shearing efficiency for all DNA samples. The sonication parameters were standardized for the fragment size requirements, including peak incident power (140 W), duty factor (10%), cycles per burst (200), and treatment time (75 s).

Fragment size distribution and quality of the sheared DNA were assessed using the Agilent 2,100 Bioanalyzer with a High Sensitivity DNA Kit (Agilent, Cat #5067-4626). Samples exhibiting the target fragment range and a uniform distribution without degradation were selected for subsequent sample preparation.

#### 2.4.2 Bisulfite conversion

Following purification, bisulfite conversion was carried out using the EZ DNA Methylation-Gold™ Kit (Zymo Research, cat #D5005), following the manufacturer's protocol. This treatment deaminates unmethylated cytosines to uracil, while methylated cytosines remain unconverted, thereby preserving methylation information. The bisulfite-converted DNA was processed through the provided column in 20 μL of DD water for downstream library preparation.

#### 2.4.3 Library preparation

Bisulfite-converted DNA was used for library preparation. Libraries were generated using the xGen™ Methyl-Seq DNA Library Prep Kit (Integrated DNA Technologies, Cat #10009860) following the manufacturer's protocol, optimized for low-input and bisulfite-treated DNA. Approximately 100 ng of converted DNA was used per sample. The workflow included an initial Adaptase™ step to add a partial adapter sequence to single-stranded DNA, followed by an extension reaction to synthesize the complementary strand. A post-extension cleanup was performed using AMPure XP magnetic beads (Beckman Coulter, Cat # A63880). This was followed by ligation of methylated adapters, then a post-ligation cleanup using AMPure XP beads. Libraries were then amplified via indexing PCR using a uracil-tolerant polymerase, followed by a post-indexing PCR cleanup using AMPure XP magnetic beads to remove excess primers and small fragments. Final libraries were quantified using the Qubit dsDNA HS Assay Kit and used for downstream hybridization capture.

#### 2.4.4 Hybridization capture

The libraries prepared above were pooled in equal mass amounts and subjected to target enrichment using the xGen™ Custom Hybridization Capture Panel (Integrated DNA Technologies), following the manufacturer's protocol (41). The pooled library was mixed with a custom-designed probe panel targeting 25 genomic regions across 10 genes implicated in serotonin signaling and cardiac function.

A targeted candidate-gene approach was used to evaluate DNA methylation rather than an unbiased genome-wide discovery strategy. Candidate genes were selected *a priori* based on their relevance to serotonergic signaling and cardiac calcium handling, two biological pathways relevant to sertraline exposure and cardiac development and function. Gene selection was informed by published evidence and further supported by our previous transcriptomic and functional studies using the same experimental model, which identified alterations in these pathways following perinatal sertraline exposure.

Within each candidate gene, capture probes were designed to target promoter regions and other regulatory elements, with most targeted sequences located within 2 kb of the transcription start site (TSS). This design enabled focused assessment of DNA methylation within predefined candidate regulatory regions.

The targeted genes included *Slc6a4* (serotonin transporter), *Tph2* (tryptophan hydroxylase 2), *Htr2a*, *Htr2b*, and *Htr4* (serotonin receptors), *Slc8a1* (sodium-calcium exchanger 1, *NCX1*), *Pln* (phospholamban), *Ryr2* (ryanodine receptor 2), *Atp2a2* (*Serca2*), and *Tnnc1* (cardiac troponin C). Target regions were selected to include gene promoters and CpG-rich regulatory elements, typically within 2 kb of the transcription start site (TSS). Capture probes were designed and synthesized using the xGen™ Custom Panel Designer.

Hybridization was carried out for 4–16 h to allow specific binding between probes and target DNA fragments. Following hybridization, probe-bound DNA was captured using streptavidin-coated magnetic beads and subjected to a series of washes to remove nonspecifically bound sequences. Captured DNA was eluted and subsequently amplified by post-capture PCR using a uracil-tolerant polymerase (KAPA HiFi HotStart Uracil + ReadyMix Kit, Roche, Cat. #KK2801). Final libraries were purified with two rounds of AMPure XP magnetic bead cleanup to remove residual contaminants, quantified using the Qubit dsDNA HS Assay Kit, and assessed for quality and fragment integrity using the Agilent Bioanalyzer (High Sensitivity DNA Kit) prior to sequencing.

#### 2.4.5 Sequencing and bioinformatics analysis

Captured libraries were sequenced on the Illumina MiSeq platform using a MiSeq Reagent Kit v3 (300 cycles), generating paired-end 150 bp reads with dual indexing. Approximately 12% of reads aligned to the PhiX control genome, consistent with its use as a sequencing quality control spike-in. Sequencing was performed by the Genomics Division of the Iowa Institute of Human Genetics (IIHG).

Raw sequencing reads were processed by the Bioinformatics Division of IIHG using the nf-core/methylseq pipeline (version 3.0.0) implemented in Nextflow (version 24.10.5) within a Singularity container on the Argon High Performance Computing (HPC) cluster. Libraries were run in non-directional mode, and the pipeline performed adapter and low-complexity end trimming (10 bp, as recommended for xGen Methyl-Seq libraries), quality control, alignment of bisulfite-converted reads to the mouse reference genome (GRCm38) using Bismark (version 0.24.2), CpG methylation calling. All subsequent coverage filtering, normalization, and differential methylation analyses were performed in R using methylKit (version 1.32.1).

Target regions were selected using the Ensembl Release 112 Regulatory Features track, together with gene and transcript annotations from Ensembl and Rat Genome Database, to identify predefined regulatory regions within the candidate genes. Regions proximal to annotated transcription start sites (TSSs) were selected as promoter-associated targets, while regions annotated as candidate enhancers in the Ensembl Regulatory Features track were selected as enhancer targets. Capture probes were designed to target these predefined regions. The target regions were originally defined using the GRCm39 mouse genome assembly, and probe coordinates were subsequently converted to GRCm38 using the UCSC liftOver chain to ensure compatibility with the alignment reference genome.

Following methylation calling, CpG methylation was quantified using methylKit. To improve the reliability of methylation estimates and reduce the influence of low-coverage sites, CpG sites with fewer than 25 reads in any sample were discarded at the data-import step. Sites exceeding the 99.9th percentile of per-sample coverage were removed to exclude potential PCR-duplication artifacts, and read coverage was median-normalized across samples. Because the libraries were non-directional and no strand information was retained, CpG sites were not destranded. Only cytosines covered in all eight samples were retained, ensuring complete methylation measurements across samples.

##### 2.4.5.1 Region-level differential methylation was the primary analysis

Read counts of covered CpGs within each of the 25 predefined targeted regions were aggregated, and regions with at least three covered cytosines in every sample were retained. Differential methylation between saline- and sertraline-treated groups was evaluated using logistic regression in methylKit with overdispersion correction using the MN method and an *F*-test. This approach accounts for between-animal biological variability rather than treating individual sequencing reads as independent observations. Multiple testing was controlled using the Benjamini–Hochberg (BH) procedure, with adjusted *q* values <0.05 considered statistically significant. No inter-sample methylation SD filter was applied to the primary regional analysis.

##### 2.4.5.2 Site-level differential methylation was evaluated separately as a secondary sensitivity analysis

Individual CpG sites were analyzed using two inter-sample methylation variability thresholds: SD >2% as the primary site-level sensitivity analysis and the more permissive SD >1% threshold as an additional sensitivity analysis. These filters were applied only to the site-level analyses. Differential methylation was assessed using the same overdispersion-corrected model with a *χ*2 test, followed by BH adjustment. Given the four biological replicates per group, site-level analyses were considered exploratory and hypothesis-generating rather than confirmatory.

### 2.5 Statistical analysis

Data are presented as mean ± SEM. Statistical analyses of animal-level measurements and ELISA-based global DNA methylation data were performed using GraphPad Prism 10 (GraphPad Software, Boston, MA, USA). Normality was assessed using the Shapiro–Wilk test. For comparisons in which one or both groups were not normally distributed, the Mann–Whitney *U* test was used. A two-sided *p* value < 0.05 was considered statistically significant, with *p* < 0.01 indicating stronger statistical significance. Sex was considered during experimental design and was evaluated as a potential source of variation in the methylation data.

## 3 Results

### 3.1 Sertraline exposure and offspring monitoring

Five pregnant dams per group were monitored throughout gestation and lactation for changes in body weight, and the body weights of their offspring were recorded daily until PN14, when pups were euthanized for heart collection. These outcomes were consistent with our previous findings and are detailed in Supplementary Figures S1–S4. From each group, heart samples from four pups per group were randomly selected for TBS-seq analysis. Additional randomly selected samples were used for global 5-mC quantification by ELISA.

### 3.2 Global 5-mC levels

Global 5-mC levels in PN14 mouse hearts were assessed using a colorimetric ELISA, and the results are shown in Figure 1. No statistically significant difference was detected between the sertraline-exposed group (1.83 ± 0.19%, *n* = 16) and saline controls (2.55 ± 0.38%, *n* = 16) (*p* > 0.05), suggesting that sertraline exposure did not significantly alter global 5-mC levels in the heart.

**Figure 1:**
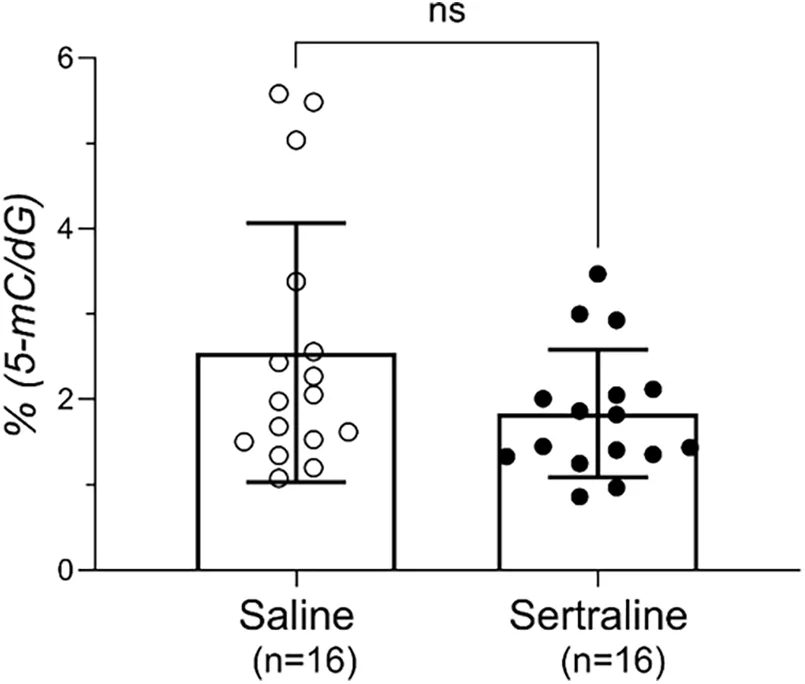
Global 5-mC levels in PN14 mouse hearts following developmental sertraline exposure. Global 5-methylcytosine (5-mC) levels were measured in cardiac DNA from postnatal day 14 (PN14) mice. Data are shown as individual values with mean ± SEM. Normality testing using the Shapiro–Wilk test indicated a non-normal distribution in the saline control group (*p* < 0.05). A Mann–Whitney *U* test was used to compare groups (*p* = 0.1835).

### 3.3 Regional DNA methylation profiling by TBS-seq

Eight DNA samples from left ventricular tissues of PN14 mice were analyzed, comprising four biological replicates from saline-treated controls (*n* = 4) and four from sertraline-exposed mice (*n* = 4). The sheared DNA fragments were at the desired size range centered around 300–500 bp as shown in the unimodal, bell-shaped profiles of electropherogram traces obtained using Agilent 2,100 Bioanalyzer (Supplementary Figure S5).

A Browser Extensible Data (BED) file defining the target regions was generated using the FAIR (Findable, Accessible, Interoperable, and Reusable resource) online platform (47). Based on published evidence and our previous studies, ten genes were selected for analysis, five in serotonin signaling (*Htr2a*, *Htr2b*, & *Htr4*, *Slc6a4*, and *Tph2*) and five in cardiac calcium handling (*Atp2a2*, *Pln*, *Ryr2*, *Slc8a1*, and *Tnnc1*) (25). Custom-designed capture probes were generated by IDT, and used to target 25 regulatory regions across these genes (Supplementary Table S1).

High-quality, final library was obtained from all samples and subjected to hybridization capture (Supplementary Figure S6), followed by paired-end Illumina sequencing and downstream bioinformatics analysis. Subsequent analyses focused on CpG methylation patterns within the captured target regions.

#### 3.3.1 TBS-seq yield and quality assessment

All samples were processed using the same TBS assay and sequencing batch. TBS-seq was performed on an Illumina MiSeq platform, yielding −13.1 million paired-end reads, with 12% aligning to the PhiX control genome. Approximately 85.6% of reads passed quality filtering, resulting in −11.5 million high-quality reads. The initial step of the bioinformatics analysis employed a standardized and reproducible Nextflow pipeline (v24.10.5, build 5,935) to process raw paired-end TBS-seq data.

At the sequencing level, unique alignment rates ranged from 74.6% to 77.5% across the eight samples, indicating comparable alignment performance between samples (Supplementary Table S3). Mean target coverage ranged from 144 × to 204 × across individual samples (Supplementary Table S3).

A minimum coverage threshold of >25 × was applied to improve the reliability of CpG-level methylation estimates and reduce the influence of low-coverage sites. Before coverage filtering, 9,349 CpG sites were identified in the merged dataset, defined as sites with at least one read across the eight samples. Following the >25 × coverage filter, 1,941 CpG sites remained for subsequent on-target and regional quality filtering across the 25 predefined target regions. Of these regions, 23 met the predefined criterion of having at least three covered cytosines per region in every sample, resulting in 1,411 CpG sites across the 23 retained regions. Among these CpG sites, 619 showed an inter-sample methylation standard deviation (SD) >1%, and 414 showed an SD >2% (Supplementary Figure S7).

All samples exhibited a unimodal coverage distribution, with most CpG sites showing approximately 10^1.5^–10^2.5^ reads per base (>25×) across the 1,941 captured CpG sites (Supplementary Figure S8A). The 1,411 CpG sites within the retained target regions were subsequently used for coverage-quality assessment and comparative analyses between the saline control and sertraline-exposed groups.

Across the predefined target regions, per-base coverage ranged from 49 × to 378 × . Mean coverage was comparable between the saline control group (176 ± 2 × ) and the sertraline-exposed group (173 ± 2 × ) (Supplementary Figure S8B), with only a modest difference between groups. The comparable coverage distributions indicate no substantial group-level difference in sequencing depth that would be expected to introduce a major technical bias into downstream methylation comparisons.

#### 3.3.2 Overall methylation profiles and inter-animal variation

To characterize the overall DNA methylation landscape within the captured regions, we examined the distribution of CpG methylation percentages across the 1,411 targeted CpG sites within 23 regions in the eight samples (Figure 2A). Across individual samples, methylation profiles exhibited a unimodal distribution, with a prominent peak in the >0%–10% methylation bin. Approximately 52 ± 1% of CpG sites per sample were hypomethylated (0%–10%), whereas each of the remaining bins from 20% to 100% methylation contributed modestly (2%–8%) to the overall distribution. This distribution differs from the bimodal methylation patterns commonly reported in genome-wide methylation studies, potentially reflecting differences in sequencing strategy and the predefined regulatory regions targeted in the present study (48).

**Figure 2:**
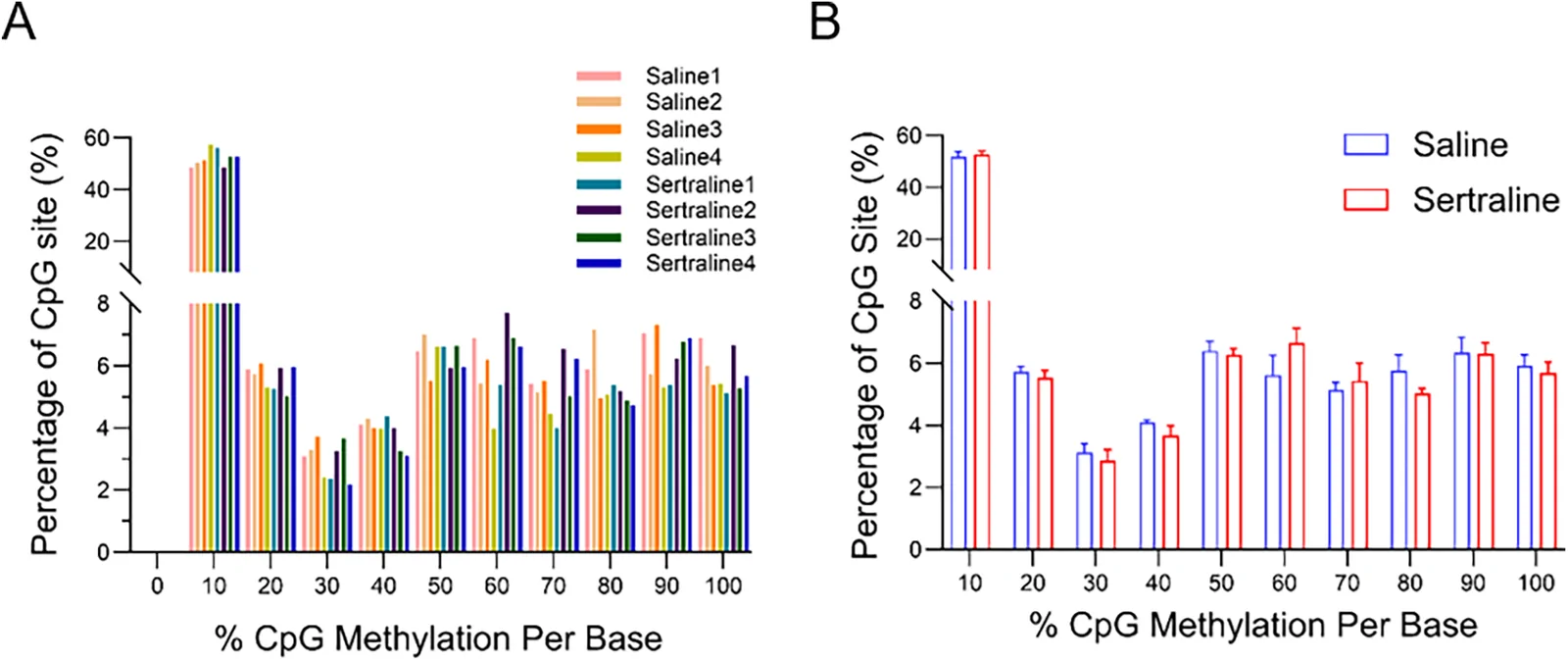
Frequency distribution of CpG methylation levels across bases of 1,411 sites within 23 regions in the eight samples. **(A)** Distribution of CpG methylation levels in individual samples. Each line represents one sample, with light-colored lines indicating saline-exposed samples (*n* = 4) and dark-colored lines indicating sertraline-exposed samples (*n* = 4). Unmethylated CpG sites (methylation = 0%) were excluded from the visualization to improve resolution of the distribution among methylated sites. **(B)** Group-level distributions for saline and sertraline samples, showing mean methylation profiles ± SEM across the targeted CpG sites and regions. A two-sample Kolmogorov–Smirnov test comparing the global cumulative distribution functions showed no significant difference between the saline and sertraline groups (*p* = 0.9269, D = 0.0206).

Across the 1,411 retained CpG sites, 650 sites showed higher methylation and 633 sites showed lower methylation in the sertraline group relative to the saline group, while 128 sites showed minimal or neutral methylation differences, indicating no predominant directional bias toward hypermethylation or hypomethylation.

At the group level, the overall methylation distributions of the saline and sertraline groups were highly similar, with substantial overlap between the mean methylation profiles (Figure 2B). Consistent with this observation, violin plots showed similar distributions of global CpG methylation percentages between groups (Supplementary Figure S12). Statistical comparison using the Mann–Whitney *U* test detected no significant difference in global CpG methylation between groups (*p* > 0.05). Similarly, cumulative distribution analysis revealed no significant global shift in methylation levels (Supplementary Figure S13; Kolmogorov–Smirnov test, D = 0.0206, *p* = 0.9269).

To further evaluate inter-animal variability, pairwise Pearson correlation analysis and principal component analysis (PCA) were performed using methylation levels across the targeted CpG sites. Pairwise correlations were high across all samples, with Pearson correlation coefficients (*r*) ranging from 0.97 to 0.98 (Supplementary Figure S9). Intra-group correlations were slightly higher than inter-group correlations, indicating modest differences in overall methylation profiles between treatment groups. PCA showed some separation according to treatment group, with PC1 explaining 20.3% of the total variance (Supplementary Figure S10). S3 (saline) and S8 (sertraline) showed greater deviation from their respective group centroids, but z-score and Mahalanobis distance assessment indicated that all samples remained within the predefined acceptable thresholds (Supplementary Figure S11). All samples were therefore retained for downstream analyses, and no obvious batch-related clustering was observed.

#### 3.3.3 Genomic characteristics of the targeted regions

To further characterize the genomic context of the analyzed CpG sites, we examined their distribution relative to transcription start sites (TSSs) and across annotated genomic features. Among the 1,411 CpG sites retained from the high-quality TBS-seq dataset, most were located within promoter-proximal regions surrounding the TSS (Figure 3). Within the core promoter-proximal region (−500 to +500 bp), CpG sites showed a relatively balanced distribution immediately upstream and downstream of the TSS, with 160 sites located at −100 to 0 bp and 165 sites at 0 to 100 bp. The highest local enrichment was observed at 100–300 bp downstream of the TSS (*n* = 239). Additional CpG sites were distributed at more distal positions upstream and downstream of the TSS, including loci extending beyond ±2 kb.

**Figure 3:**
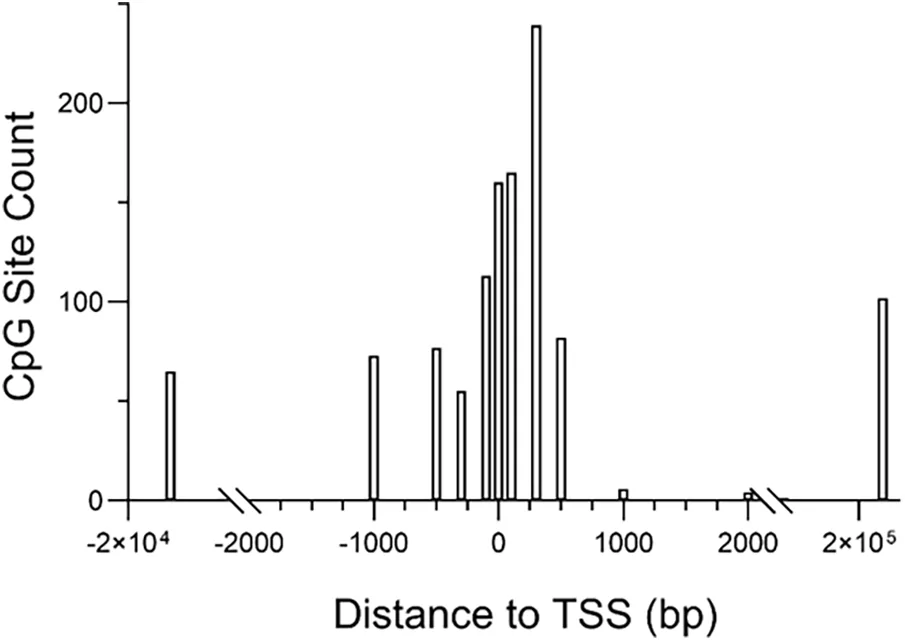
Genomic distribution of targeted CpG sites relative to transcription start sites (TSSs). The positional distribution of 1,411 CpG sites within 23 targeted regions is shown according to their distance from the nearest TSS. Most CpG sites were located within promoter-proximal regions surrounding the TSS. Within the core promoter-proximal region (−500 to +500 bp), CpG sites were most densely distributed immediately downstream of the TSS, with the highest density observed at 100–300 bp downstream (*n* = 239). The distribution was relatively balanced immediately upstream and downstream of the TSS, with 160 CpG sites at −100 to 0 bp and 165 sites at 0 to 100 bp. Additional CpG sites were distributed at more distal positions upstream and downstream of the TSS, including distal intergenic loci extending beyond ±2 kb.

Consistent with their positional distribution relative to TSSs, genomic feature annotation showed that most CpG sites were located in promoter-proximal regions, with 82.7% within 1 kb and an additional 5.4% within 1–2 kb of the TSS (Figure 4). Distal intergenic regions accounted for 8.6% of the CpG sites, whereas smaller proportions were located in intronic regions and 3′ untranslated regions (UTRs). This distribution reflects the targeted design of the TBS-seq panel, which was enriched for promoter-proximal and other regulatory genomic regions.

**Figure 4:**
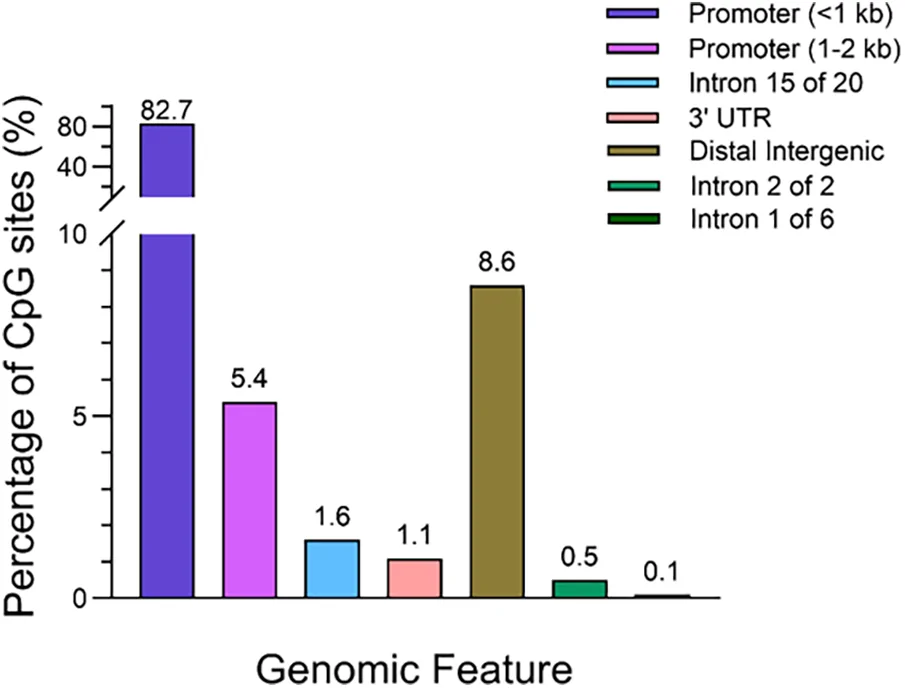
Genomic feature distribution of analyzed CpG sites. The percentage distribution of the 1,411 analyzed CpG sites across annotated genomic features is shown. Most CpG sites were located in promoter-proximal regions, with 82.7% within 1 kb and an additional 5.4% within 1–2 kb of the TSS. Distal intergenic regions accounted for 8.6% of CpG sites, while smaller proportions were located in intronic regions and 3′ untranslated regions (UTRs). The distribution reflects the targeted design of the TBS-seq panel, which was enriched for promoter-proximal and other regulatory regions.

#### 3.3.4 Regional differential DNA methylation

The primary differential methylation analysis was conducted at the regional level using the full high-quality dataset, without applying SD-based filtering. Methylation levels across CpG sites within each of the 23 predefined genomic regions were aggregated and compared between the saline (*n* = 4) and sertraline (*n* = 4) groups to identify regional differences associated with sertraline exposure. Regional differential methylation was assessed using logistic regression with Williams' method for overdispersion correction, followed by Benjamini–Hochberg (BH) adjustment for multiple testing. The complete results for all 23 targeted regions are summarized in Table 1.

**Table 1:** Targeted genomic regions with differential DNA methylation between saline- and sertraline-treated groups.

No.	ID	Chr	Start	End	Meth. Diff	*p*-value	*q*-value
1	*Htr2a*_enhancer1	14	74,651,360	74,651,960	8.19298068	4.0837 × 10^−15^	9.3924 × 10^−14^
2	*Tph2*_associated region	10	115,076,895	115,078,995	−3.00306226	0.00198413	0.02281754
3	*Tph2*_enhancer	10	115,184,095	115,185,295	1.12697248	0.11160117	0.740113
4	*Tnnc1*_promoter1	14	31,206,543	31,208,643	1.42347516	0.24029101	0.740113
5	*Slc8a1*_CpG	17	81,670,171	81,670,621	0.05849927	0.30831663	0.740113
6	*Htr4*_promoter2	18	62,322,429	62,324,529	1.31960779	0.3432488	0.740113
7	*Tph2*_promoter2	10	115,184,895	115,185,295	0.55551429	0.36103472	0.740113
8	*Slc6a4*_promoter1	11	77,009,174	77,009,574	0.97465941	0.3820879	0.740113
9	*Htr2b*_promoter3	1	86,096,278	86,099,078	0.58860203	0.38918039	0.740113
10	*Atp2a2*_CpG	5	122,501,237	122,502,787	0.03247769	0.38961247	0.740113
11	*Ryr2*_promoter2	13	11,551,314	11,553,414	1.04455855	0.39134695	0.740113
12	*Slc8a1*_promoter	7	82,028,752	82,031,352	0.51219466	0.43002653	0.740113
13	*Htr2a*_promoter1	14	74,639,060	74,641,160	−1.07728826	0.46800898	0.740113
14	*Slc8a1*_enhancer	17	81,385,771	81,386,271	−1.22392906	0.47839141	0.740113
15	*Pln*_promoter1	10	53,337,504	53,337,904	2.67903917	0.48988394	0.740113
16	*Ryr2*_CpG	13	12,105,914	12,107,064	0.03277089	0.56474649	0.740113
17	*Pln*_promoter2	10	53,335,904	53,338,004	1.36701895	0.56498975	0.740113
18	*Slc6a4*_promoter2	11	76,996,874	76,998,974	−0.09757912	0.58848532	0.740113
19	*Atp2a2*_promoter1	5	122,451,737	122,453,837	0.5213556	0.63833122	0.740113
20	*Htr2a*_CpG	14	74,732,160	74,732,810	0.0101769	0.64357652	0.740113
21	*Htr4*_enhancer	18	62,325,529	62,325,929	−0.91328511	0.76637465	0.8052962
22	*Tph2*_promoter1	10	115,175,595	115,176,095	−0.4044793	0.79150758	0.8052962
23	*Htr2b*_enhancer1	1	86,111,878	86,112,478	−0.27376346	0.8052962	0.8052962

The 23 targeted regions were defined according to the BED file, with a minimum requirement of three covered cytosines per region in every sample. Δ Methylation (%) represents the difference in mean regional methylation between the sertraline and saline groups, with positive and negative values indicating higher and lower methylation in the sertraline group, respectively. *p*-values are unadjusted, whereas *q*-values were calculated using the Benjamini–Hochberg (BH) procedure for false discovery rate (FDR) adjustment. Genomic coordinates are based on the GRCm38 reference genome.

At the regional level, two of the 23 targeted regions showed significant differential methylation after FDR correction (*q* < 0.05) (Table 1; Figure 5). The overall distribution of regional methylation differences is shown in the volcano plot in Supplementary Figure S14A, while the corresponding methylation patterns across individual samples for the top 10 ranked target regions are illustrated by the heatmap in Supplementary Figure S14B. These analyses highlighted the *Htr2a* enhancer and *Tph2-*associated region as the two regions with statistically significant differential methylation.

**Figure 5:**
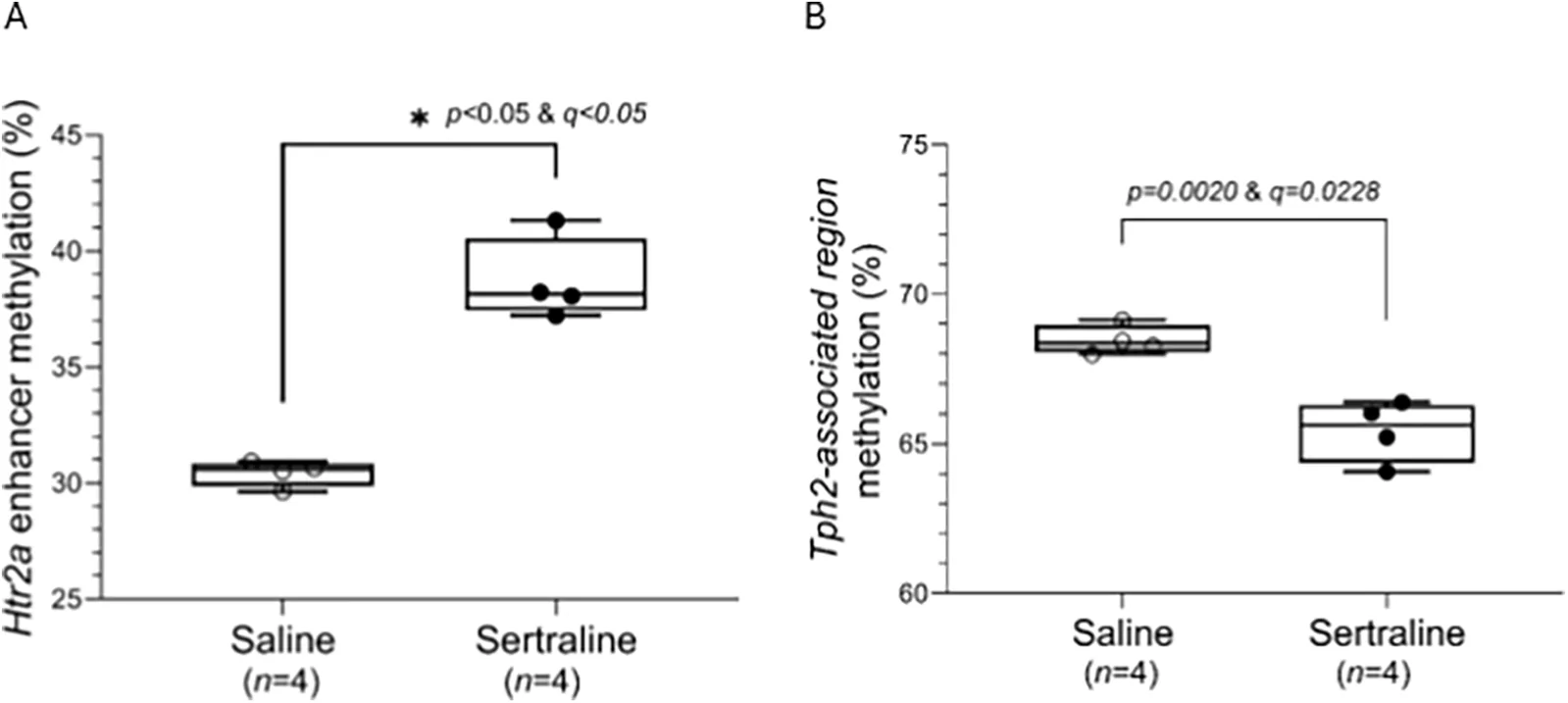
Regional-level DNA methylation analysis reveals two significantly altered genomic regions. To perform region-level evaluation, the methylation statuses of 1,411 CpG sites across 8 samples were aggregated. A total of 23 genomic regions met the inclusion criteria of having at least three covered cytosines per region in every sample. Differential methylation between the saline (*n* = 4) and sertraline (*n* = 4) groups was evaluated using logistic regression with Williams’ method (MN correction) for overdispersion, followed by the Benjamini–Hochberg (BH) procedure for multiple-testing correction. Statistical significance was defined as a BH-adjusted *q*-value <0.05. **(A)** Methylation level at the *Htr2a* enhancer region (*p* = 4.1 × 10^−15^, *q* = 9.4 × 10^−14^). **(B)** Methylation level at the *Tph2-*associated region (*p* = 0.0020, *q* = 0.0228).

The *Htr2a* enhancer region showed increased methylation in the sertraline group, with mean regional methylation 8.19 percentage points higher than that in the saline group (Δ methylation = + 8.19%; *p* = 4.08 × 10⁻^15^, *q* = 9.39 × 10⁻^14^; Figure 5A). In contrast, methylation at the *Tph2*-associated region was 3.00 percentage points lower in the sertraline group (Δ methylation = −3.00%; *p* = 0.00198, *q* = 0.0228; Figure 5B). The remaining 21 targeted regions did not show statistically significant differential methylation after FDR correction.

#### 3.3.5 CpG-level sensitivity analysis

To further assess the robustness of the regional differential methylation findings, we performed a secondary site-level analysis of individual CpG sites using the 1,411 retained CpGs. This analysis was conducted separately from the primary regional analysis and evaluated as a sensitivity analysis using two inter-sample methylation variability thresholds. Among the 1,411 CpG sites, 619 showed an inter-sample methylation SD >1%, whereas 414 showed an SD >2%. The SD >2% analysis was used as the primary site-level sensitivity analysis, while the more permissive SD >1% threshold was evaluated as an additional sensitivity analysis.

Using the CpGs with SD >2%, none of the tested sites reached statistical significance after Benjamini–Hochberg (BH) correction (all *q* > 0.05; Supplementary Table S5). The same pattern was observed in the SD >1% sensitivity analysis, with no individual CpG site remaining significant after multiple-testing correction (all *q* > 0.05; Supplementary Table S4). Although several CpG sites showed nominal *p*-values <0.05, these associations did not remain significant after FDR adjustment.

Thus, the two FDR-significant findings identified in the present study were detected in the primary regional analysis using the full high-quality dataset without SD-based filtering, whereas the site-level analyses were performed separately as sensitivity analyses and did not identify any FDR-significant individual CpG sites. The regional findings were based on aggregated methylation across CpG sites within predefined genomic regions and therefore provide a complementary level of analysis to the site-specific results.

For transparency and to preserve the complete site-level methylation dataset, methylation values for all 1,411 retained CpG sites without SD-based filtering are also provided online (https://doi.org/10.6084/m9.figshare.32509689.v2). This complete dataset enables evaluation of CpG-level methylation patterns without imposing an additional variability-based filtering criterion.

## 4 Discussion

The present study provides evidence that the epigenetic response to perinatal sertraline exposure in the developing heart may be characterized by localized changes across regulatory regions rather than uniform or highly focal changes at individual CpG sites. This distinction is important because methylation changes within regulatory elements can involve coordinated or heterogeneous changes across multiple CpGs, such that the biological signal may become more apparent when methylation information is considered at the regional level. In the present dataset, the primary regional analysis, performed without SD-based filtering, identified exposure-associated signals that were not recapitulated as significant individual CpG sites in the secondary sensitivity analyses after correction for multiple testing. This pattern suggests that regional aggregation may provide a biologically relevant level of resolution for detecting modest epigenetic alterations in neonatal cardiac tissue, particularly when biological variability and the limited number of animals are considered.

One possible interpretation is that perinatal sertraline exposure does not produce a widespread shift in cardiac DNA methylation, but instead affects selected regulatory elements associated with specific biological pathways. The two regions identified in the primary analysis were located near genes involved in serotonergic signaling, providing a potential molecular link between pharmacological exposure and cardiac epigenetic regulation. However, the functional consequences of these methylation changes remain to be established, and their association with serotonergic signaling should therefore be considered a hypothesis for further investigation rather than evidence of a causal mechanism.

The involvement of serotonergic regulatory loci is biologically plausible because serotonin signaling contributes to cardiac development, cardiomyocyte proliferation, and ventricular maturation (18, 21, 22). The serotonin receptor 2A (*Htr2a*) is involved in serotonin-mediated cellular signaling whereas tryptophan hydroxylase 2 (*Tph2*) encodes an enzyme involved in serotonin biosynthesis. Altered methylation within regulatory regions of these genes could therefore reflect epigenetic modulation of cardiac serotonergic pathways following perinatal SSRI exposure. Importantly, however, the present study did not directly measure the transcriptional or functional consequences of methylation at these loci.

These findings can also be considered in the context of our previous observations from an independent cohort exposed under the same experimental conditions, in which perinatal sertraline exposure was associated with reduced expression of several serotonergic genes (25). Although methylation and gene expression were not measured in the same animals, the convergence of these observations provides a rationale for investigating whether the identified epigenetic alterations are associated with transcriptional changes in cardiac serotonergic pathways. Such an interpretation remains associative, however, because the present data cannot establish whether the observed methylation differences precede or result from changes in gene expression.

DNA methylation is an important component of the epigenetic regulation of cardiac development and contributes to the temporal control of gene expression during cardiomyocyte differentiation and maturation (27). Gilsbach *et al*. demonstrated that cardiac promoters and enhancers can exhibit distinct methylation states associated with tissue-specific transcriptional activity (27). The enrichment of targeted CpG sites within promoter-proximal regions in the present study is consistent with the regulatory focus of the TBS-seq panel and supports the use of targeted methylation profiling for investigating candidate cardiac regulatory elements.

Targeted bisulfite sequencing provides deep coverage of predefined genomic regions and is therefore well suited to hypothesis-driven interrogation of candidate regulatory loci (42, 43, 48, 49). Similar targeted methylation approaches have been used to investigate epigenetic alterations in cardiovascular disease and cardiac injury (50–53). In the present context, this approach allowed focused assessment of regulatory regions selected on the basis of their potential relevance to cardiac and serotonergic biology. The findings therefore complement, rather than replace, broader genome-wide approaches that may identify additional exposure-associated loci outside the targeted panel.

The absence of FDR-significant individual CpGs in the secondary analysis should be interpreted within the statistical context of the dataset. Hundreds to thousands of CpG sites were evaluated, and multiple-testing correction is intended to reduce the likelihood that nominally significant associations reflect false-positive findings. In addition, individual CpGs may exhibit substantial biological and technical variability, whereas regional analyses leverage methylation information across neighboring CpG sites, potentially increasing power to detect coordinated epigenetic variation. Taken together, these results support prioritizing interpretation of the regional findings while viewing the CpG-level analyses as exploratory and hypothesis-generating.

Notably, Non *et al*. investigated genome-wide DNA methylation in cord blood from neonates exposed to maternal depression or anxiety or SSRI use during pregnancy (54). Although differential methylation was observed in neonates exposed to maternal depression or anxiety, no CpG sites remained significantly associated with SSRI exposure after correction for multiple comparisons. Together with the absence of FDR-significant individual CpGs in the present study, these findings illustrate the challenges of detecting robust site-specific methylation changes following SSRI exposure and highlight the importance of tissue-specific and biologically informed study designs. The cardiac-targeted approach used here enables focused interrogation of selected regulatory regions that may be missed or insufficiently represented in broader analyses.

The functional significance of the identified methylation changes remains unresolved. DNA methylation is only one component of epigenetic regulation, and changes within promoters or enhancers do not necessarily result in predictable changes in transcription. Chromatin accessibility, transcription factor binding, histone modifications, and enhancer–promoter interactions may all influence the functional consequences of altered methylation. In the present study, DNA methylation was assessed in an independent cohort from that used in our previous gene expression study; therefore, direct within-animal relationships between the identified methylation changes and transcriptional phenotypes could not be established. Future studies integrating DNA methylation with transcriptomic and chromatin-accessibility measurements in the same animals, including targeted ATAC-qPCR validation of relevant loci, will be important for determining whether the identified regions are associated with altered regulatory activity and transcriptional changes.

Several limitations should be considered. First, the study included four biological replicates per treatment group, limiting statistical power and emphasizing the need for independent replication. Although the overdispersion-corrected model accounts for between-animal variability, larger cohorts will be necessary to determine the reproducibility of the identified regions. Sex was balanced between treatment groups, with two females and two males per group, and sex-related variation was evaluated in the methylation data. However, the small number of animals within each sex precludes robust assessment of sex-specific treatment effects. Second, the targeted TBS-seq design restricts the analysis to predefined genomic regions and cannot exclude methylation changes elsewhere in the genome. Third, bisulfite sequencing does not distinguish 5-methylcytosine from 5-hydroxymethylcytosine, which may be relevant in cardiac tissue (27).

The upstream mechanisms responsible for the observed methylation changes remain unresolved. In particular, DNMT and TET expression or activity was not assessed in the present study; therefore, it cannot be determined whether altered regulation of these epigenetic enzymes contributes to the observed locus-specific methylation changes. Future studies incorporating DNMT/TET profiling together with DNA methylation and gene expression measurements will be important for clarifying the molecular mechanisms linking perinatal sertraline exposure to cardiac epigenetic regulation.

The use of bulk left ventricular tissue represents another important limitation. The heart contains multiple cell populations with distinct epigenetic profiles, and differences in cellular composition or developmental state could contribute to the observed methylation patterns. Notably, sertraline-exposed pups were smaller at PN14, raising the possibility that some of the observed methylation differences may reflect differences in developmental maturity rather than direct effects of exposure on DNA methylation. Consequently, the present data cannot distinguish cell-intrinsic methylation changes from those associated with altered cellular composition. Developmental stage, or a combination of these factors. Cell-type-resolved epigenomic approaches, together with assessments at developmentally matched time points, will be necessary to address these questions.

In addition, DNA methylation was assessed only at PN14, and the developmental onset and persistence of the identified methylation changes remain unknown. Future longitudinal studies spanning embryonic and postnatal development, including assessment at E14.5, E18.5, PN0, PN7, followed by later development stages as PN 30 and adulthood, will be important for determining when these epigenetic alterations first arise, whether they persist over time, and whether they are associated with long-term cardiac outcomes.

Potential litter effects were not explicitly accounted for in some of the ancillary outcomes, including body weight, survival, heart weight, and global 5-mC analyses, because multiple pups from the same litters contributed to these assessments. For the global 5-mC analyses, tissues were randomly selected from available mouse samples and litter was not incorporated into the analysis. These findings should therefore be interpreted with appropriate caution, as litter-level influences cannot be excluded.

Overall, these findings support a model in which perinatal sertraline exposure is associated with selective alterations in cardiac regulatory regions rather than a broad global methylation shift. The *Htr2a* enhancer and *Tph2*-associated region provide candidate loci for further investigation of the relationship between serotonergic signaling, developmental epigenetic regulation, and cardiac outcomes following early-life SSRI exposure. Independent replication and functional validation will be required to determine the biological significance of these associations.

## 5 Conclusion

This study identifies localized epigenetic alterations in the neonatal heart associated with perinatal sertraline exposure and highlights cardiac serotonergic regulatory regions as potential targets of early-life SSRI exposure. These findings provide a basis for further investigation of how developmental exposure to sertraline may influence cardiac epigenetic regulation and, ultimately, cardiovascular development. However, because analyses were performed in bulk left ventricular tissue at a single postnatal time point, the observed methylation differences may reflect direct cell-intrinsic epigenetic changes, differences in cellular composition, developmental maturity, or a combination of these factors. Functional validation and independent replication will be important to determine the biological significance and persistence of these epigenetic alterations and their potential relationship to later-life cardiac outcomes.

## Data Availability

The datasets presented in this study can be found in online repositories. The names of the repository/repositories and accession number(s) can be found in the article/Supplementary Material.
