# Supplementary material for: Early epigenetic alterations in cardiac tissue following perinatal sertraline exposure in mice
Source: Front Cardiovasc Med. 2026 Oct 28;13:1894882. doi: 10.3389/fcvm.2026.1894882 (PMC13751448; doi:10.3389/fcvm.2026.1894882)
Supplement: Supplementary file 1 [file Datasheet1.docx]

**Supplementary materials**

This Supplementary Material includes 13 supplementary figures (Figures S1–S13) and 4 supplementary tables (Tables S1–S4).

1. **Sertraline Exposure Mouse Groups and Offspring Monitoring**

A total of 10 pregnant female C57BL/6J mice (~10 weeks old) were used in the mouse model of chronic sertraline exposure, with five receiving sertraline treatment (5 mg/kg) and five receiving saline as control. The initial body weights of the dams at the onset of breeding were comparable between groups (20.38 ± 0.26 g). As shown in Figure S1, body weights of pregnant mice recorded throughout gestation and lactation revealed no significant differences between the two groups, consistent with our previous findings. The average litter size across all dams was 7 pups (range: 5–9). In total, 39 pups (from 5 litters) survived in the saline group and 31 pups (from 5 litters) in the sertraline group. Although early postnatal loss appeared slightly higher in the sertraline group within the first three days, the overall survival rate did not differ significantly between groups (93% vs. 79%; p > 0.05), as shown in Figure S2.

**Figure S1. Body weights of female mice during gestation and lactation.** Sertraline-treated mice (n = 5) were compared to saline-treated controls (n = 5). Data are presented as mean ± SD. No significant differences in body weight were observed between groups at any time point.

**Figure S2. Kaplan-Meier survival curve of mouse pups from PN day 0 to PN day 14.** Comparison of survival curves of sertraline group vs saline group was done by using the Log-rank (Mantel-Cox) test (p>0.05) and the Gehan-Breslow-Wilcoxon test (p>0.05).

The pups in the sertraline group consistently exhibited significantly lower body weights compared to the saline group (Figure S3A). On PN14, the average body weight was 7.24 ± 0.07 g (n = 39) in the saline group versus 6.53 ± 0.07 g (n = 31) in the sertraline group (p < 0.0001; Figure S3B). No sex-dependent differences in body weight were observed at PN14 (p > 0.05; data not shown).

**B**

**A**

**Figure S3. Body weight of mouse pups during early postnatal development.** (A) Sertraline exposure significantly reduced pup body weights from PN0 to PN14. Data are presented as mean ± SE. Filled circles represent saline-treated pups, and open circles represent sertraline-exposed pups. Statistical significance was assessed using the Mann–Whitney U test or unpaired two-tailed t-test, depending on data normality determined by the Shapiro–Wilk test. Significant differences were observed at PN0 (p < 0.001), PN1 (p < 0.05), and PN2 (p < 0.05), with all subsequent time points showing significance at p < 0.0001.

No significant difference in absolute heart weight was observed on PN14 between sertraline- and saline-exposed pups (43.15 ± 0.65 mg vs 44.15 ± 0.77 mg; p > 0.05; Figure S4A). However, when heart weights were normalized to body weight, the heart-to-body weight ratio was significantly higher in the sertraline group due to reduced body mass (6.53 ± 0.09 mg/g vs 5.95 ± 0.07 mg/g; p < 0.0001; Figure S4B). No sex-dependent differences in heart weight were observed in either group (data not shown).

**A**

**B**

**Figure S4. Effects of sertraline exposure on heart weights in mouse pups on PN14.**
(A) Box plot showing absolute heart weights. (B) Box plot showing heart weights normalized to body weight.
Data are presented for saline-treated (black filled circles) and sertraline-treated (red open circles) groups.

2. **DNA Sample Quality Assays**

|  | Ladder | DNA-saline | DNA-sertraline |
| --- | --- | --- | --- |
| Noise | 0.3 | 0.3 | 0.3 |
| From (bp) | 50 | 40 | 40 |
| To (bp) | 7000 | 2000 | 2000 |
| % of Total |  | 98 | 99 |
| Average size (bP) |  | 438 | 419 |
| Concentration (pg/mL) |  | 4206.16 | 4086.57 |
| Molarity (pmol/L) |  | 24189.2 | 24046.3 |
| Corr. Are |  | 4145.5 | 4252.5 |
| Size distribution in CV (%) |  | 56.7 | 56.1 |

**D**

**B**

**A**

**C**

**Figure S5. Fragment size distribution and quality of representative sheared DNA samples assessed using the High Sensitivity DNA Assay on the Agilent 2100 Bioanalyzer.** (A) DNA ladder. (B) Representative sheared DNA sample from the saline group. (C) Representative sheared DNA sample from the sertraline group. (D) Table of peak characteristics. The size distribution CV (%) represents the coefficient of variation of DNA fragment sizes (in base pairs), calculated as: CV = (standard deviation / mean fragment size) x 100. Both samples show unimodal, broad, bell-shaped fragment distributions centered around 300–500 bp, consistent with efficient mechanical shearing. Minimal high-molecular-weight carryover is observed. A CV of ~56% is acceptable for downstream TBS-seq library preparation using a hybrid-capture approach.

|  | Ladder | Library |
| --- | --- | --- |
| Noise | 0.3 | 0.4 |
| From (bp) | 50 | 150 |
| To (bp) | 7000 | 850 |
| % of Total |  | 98 |
| Average size (bp) |  | 354 |
| Concentration (pg/mL) |  | 1490.54 |
| Molarity (pmol/L) |  | 6666.2 |
| Corr Are |  | 1876.1 |
| Size distribution in CV (%) |  | 19.7 |

**C**

**A**

**B**

**Figure S6. Quality of the final hybrid captured library assessed using the high sensitivity DNA assay on the Agilent 2100 Bioanalyzer.** (A) DNA ladder. (B) Final hybrid captured library. (C) Table of peak characteristics. For TBS-seq using a hybrid-capture approach, a CV of ≤ 20-25% in the final library is preferred. A low CV indicates a tight size distribution, meaning DNA fragments are relatively uniform in length. This uniformity promotes efficient hybridization to capture probes, consistent coverage across target regions, and reliable downstream quantification and methylation analysis.

| Gene No. | Gene | Region No. | Chromosome | Start | End | Region name |
| --- | --- | --- | --- | --- | --- | --- |
| 1 | *Atp2a2* | 1 | chr5 | 122589800 | 122591900 | Atp2a2_promoter1 |
|  |  | 2 | chr5 | 122639300 | 122640850 | Atp2a2_CpG |
| 2 | *Htr2a* | 3 | chr14 | 74876500 | 74878600 | Htr2a_promoter1 |
|  |  | 4 | chr14 | 74888800 | 74889400 | Htr2a_enhancer1 |
|  |  | 5 | chr14 | 74969600 | 74970250 | Htr2a_CpG |
| 3 | *Htr2b* | 6 | chr1 | 86024000 | 86026800 | Htr2b_promoter3 |
|  |  | 7 | chr1 | 86039600 | 86040200 | Htr2b_enhancer1 |
| 4 | *Htr4* | 8 | chr18 | 62488000 | 62488600 | Htr4_promoter1 |
|  |  | 9 | chr18 | 62455500 | 62457600 | Htr4_promoter2 |
|  |  | 10 | chr18 | 62465600 | 62466000 | Htr4_promoter3 |
|  |  | 11 | chr18 | 62458600 | 62459000 | Htr4_enhancer |
| 5 | *Pln* | 12 | chr10 | 53213600 | 53214000 | Pln_promoter1 |
|  |  | 13 | chr10 | 53212000 | 53214100 | Pln_promoter2 |
| 6 | *Ryr2* | 14 | chr13 | 12120800 | 12121950 | Ryr2_CpG |
|  |  | 15 | chr13 | 11566200 | 11568300 | Ryr2_promoter2 |
| 7 | *Slc6a4* | 16 | chr11 | 76900000 | 76900400 | Slc6a4_promoter1 |
|  |  | 17 | chr11 | 76887700 | 76889800 | Slc6a4_promoter2 |
| 8 | *Slc8a1* | 18 | chr17 | 81977600 | 81978050 | Slc8a1_CpG |
|  |  | 19 | chr17 | 81693200 | 81693700 | Slc8a1_enhancer |
|  |  | 20 | chr7 | 81678500 | 81681100 | Slc8a1_promoter |
| 9 | *Tph2* | 21 | chr10 | 115011500 | 115012000 | Tph2_promoter1 |
|  |  | 22 | chr10 | 115020800 | 115021200 | Tph2_promoter2 |
|  |  | 23 | chr10 | 115020000 | 115021200 | Tph2_enhancer |
|  |  | 24 | chr10 | 114912800 | 114914900 | Tph2_associated region |
| 10 | *Tnnc1* | 25 | chr14 | 30928500 | 30930600 | Tnnc1_promoter1 |

3. **BED File and Targeted Regions**

**A Browser Extensible Data (BED) file defining the target regions was generated using the FAIR (Findable, Accessible, Interoperable, and Reusable resource) online platform. Based on prior studies, ten genes were selected for analysis, five in serotonin signaling (*Htr2a*, *Htr2b*, & *Htr4*, *Slca64*, and *Tph2*) and five in cardiac calcium handling (*Atp2a2*, *Pln*, *Ryr2*, *Slc8a1*, and *Tnnc1*). The BED file, detailed in Table S1, was submitted to Integrated DNA Technologies (IDT) for the design of custom-designed capture probes targeting 25 regulatory regions across these genes. Genomic coordinates were submitted in GRCm39/mm39 as required by IDT and converted to GRCm38/mm10 in downstream bioinformatics analysis.**

**Table S1. BED file of targeted regions of 10 genes (GRCm39/mm39)**

***Slc6a4*: Serotonin transporter, SERT.**

***Tph2*: Tryptophan hydroxylase 2, crucial for serotonin (5-HT) synthesis. TPH2 expression is detected in early embryonic tissues, including neural crest derivatives and regions that contribute to cardiac innervation.**

**5-HT receptors: *Htr2a*, *Htr2b*, *Htr4*.**

***Atp2a2*: Sarco(endo)plasmic reticulum calcium-ATPase 2 (*Serca2*), crucial for regulating calcium ion levels within cells.**

***Pln*: Phospholamban, regulating calcium transport in heart muscle cells.**

***Ryr2*: Ryanodine receptor 2, crucial for regulating calcium release in heart muscle cells.**

***Slc8a1*: Sodium-Calcium Exchanger 1, Ncx1.**

***Tnnc1*: Cardiac troponin C (*cTnC*), crucial for regulating muscle contraction in both cardiac and slow skeletal muscle.**

4. **Quality Control**

**4.1 Data Filtering**

Captured libraries were sequenced on the Illumina MiSeq platform using a MiSeq Reagent Kit v3 (300 cycles), generating paired-end 150 bp reads with dual indexing, with 12% aligning to the PhiX control genome. PhiX (ΦX174) is a small, well-characterized bacteriophage genome, consistent with typical spike-in for quality control. TBS-seq generated approximately 11.5 million high-quality paired-end reads.

To focus the analysis on biologically informative regions, a two-step filtering process was applied.

First, read counts were aggregated across all covered CpG sites. Only CpGs located within the 25 probe-defined target regions specified in the BED file were retained. Within these targeted regions, regions were required to contain at least three covered cytosines in every sample. Applying this coverage criterion and filtering based on genomic location yielded a final set of 1,411 on-target CpG sites in 23 regions for comparative methylation and coverage analyses.

Second, CpG sites were filtered based on inter-sample variability in methylation levels. Before applying the SD filter, 1,411 CpG positions were retained. Applying an SD threshold of >1% reduced this number to 619 CpG positions, while a more stringent threshold of >2% resulted in 414 CpG positions. The SD threshold was used to exclude low-variability sites that were less likely to exhibit meaningful differences between groups. The distribution of SD values across all CpG sites is shown in Figure S7.

**Figure S7. Histogram of inter-sample standard deviation (SD) of CpG methylation levels across all samples (n = 8).** The X-axis represents SD bins (in %), and the Y-axis shows the number of CpG sites within each SD range.

**4.2 Coverage Quality**

For sequencing coverage assessment to characterize the overall DNA methylation landscape, we plotted the distribution of % CpG methylation across all 1,941 covered CpG sites (Figure S8A). Subsequently, analysis was restricted to 1,411 on-target CpG sites in captured 23 regions, those located within predefined genomic regions of interest (e.g., target genes, promoters, or specific chromosomes) as specified in the BED file (Figure S8B).

For downstream analyses, only on-target CpG sites were retained to ensure biological relevance and consistency with probe design.

A	B

**Figure S8.** **Coverage quality and distribution of CpG sites from targeted bisulfite sequencing (TBS-seq).** (A) Coverage distribution across captured 1941 CpG sites. The X-axis shows log₁₀-transformed read coverage per base, and the Y-axis indicates the relative frequency (%) of CpG sites at each coverage level, normalized per sample. Light-colored lines represent saline-exposed samples (*n* = 4); dark-colored lines represent sertraline-exposed samples (*n* = 4). All samples displayed a unimodal distribution, with most CpG sites covered at ~ 10^1.5^ to 10^2.5^ reads per base (> 25x). (B) Violin plots illustrate the per-base sequencing coverage depth (reads per CpG site) obtained from 1411 CpG sites filtered by the BED-defined 23 target regions. Median coverage is indicated by green lines, and the first and third quartiles are shown as pink lines. The mean sequencing coverage depths were 176 ± 2 for the saline group and 173 ± 2 for the sertraline group, reflecting high and consistent coverages across samples (ranging from 49x to 378x). The slight difference between groups falls within expected technical variation and does not impact downstream methylation analysis.

**4.3 Pairwise Pearson Correlation of CpG Methylation**

**Figure S9. Pairwise Pearson correlation of CpG methylation levels across all TBS-seq samples (*n* = 8). The upper triangle shows Pearson correlation coefficients (*r*) between each sample pair. The diagonal panels display methylation distributions for individual samples. The lower triangle contains 2D density scatter plots with regression lines, visualizing pairwise methylation relationships. High inter-sample correlations (r ≈ 0.97–0.98) indicate strong consistency. Samples S1–S4: saline controls; S5–S8: sertraline-treated.**

**To assess global similarity in DNA methylation profiles, pairwise Pearson correlation analysis was performed using CpG methylation levels across eight heart samples: four saline-treated controls (S1–S4) and four sertraline-treated mice (S5–S8). The results were visualized using a customized pairwise scatterplot matrix (Figure S9).**

**The upper triangle of the matrix shows Pearson correlation coefficients (*r*) to four decimal places, revealing strong inter-sample correlations ranging from 0.97 to 0.98. These values indicate highly similar methylation profiles across all samples, with intra-group correlations (within saline or sertraline) slightly higher than inter-group correlations, suggesting subtle treatment-related differences.**

**The diagonal panels display the methylation value distributions for each sample. All samples exhibit similar distribution shapes, predominantly featuring CpG sites with moderate-to-high methylation levels.**

**The lower triangle presents 2D density scatter plots for each sample pair. Blue regression lines highlight strong linear relationships, while the dispersion of points provides visual insight into sample variability. Notably, samples S1 and S8 show greater deviation from their group centers in some pairwise plots, suggesting increased inter-individual variability or potential outlier characteristics.**

**These results confirm that DNA methylation profiles are highly consistent across all samples, as expected from a shared tissue type (heart). While sertraline treatment does not produce dramatic global shifts in methylation, slightly reduced inter-group correlations support subtle treatment-related epigenetic alterations. The distinct behavior of S1 and S8 warrants further exploration, though they do not meet statistical outlier thresholds.**

**4.4 CpG Methylation** **Principal Components Analysis (PCA)**

Principal component analysis (PCA) was conducted on DNA methylation data from 1,141 targeted CpG sites across 8 heart samples. The first two principal components, PC1 and PC2, accounted for 20.3% and 18.1% of the total variance, respectively (Figure S10), capturing the most prominent methylation differences across samples.

The PCA revealed partial separation between the sertraline-treated and saline control groups, primarily along PC1, suggesting treatment-associated epigenetic differences. Samples S3 (saline) and S8 (sertraline) appeared visually distant from their respective group centroids, indicating greater inter-individual variability in methylation patterns. However, these samples did not exceed standard statistical thresholds for outlier detection (|z-score| < 2.5; Mahalanobis distance < 7.8147 across the first three PCs) (Figure S11), and thus were retained for downstream analyses to preserve statistical power.

No evidence of a batch effect was observed.

**Figure S10. PCA plot of CpG methylation levels across all eight samples.** Each point represents an individual sample and is colored according to treatment group (red open circles: saline; blue filled circles: sertraline). PC1 and PC2 together explain 38.4% of the total variance. The partial separation of samples along PC1 indicates differences in methylation profiles between the treatment groups. Samples S3 and S8 are located farther from their respective group centroids, indicating greater within-group variability.

A	B

**Figure S11. Statistical assessment of sample outliers. (A) Heatmap of z-scores for PC1 and PC2 shows all samples within |z| < 2.5, indicating no univariate outliers. (B)** **Mahalanobis distance analysis based on the first two principal components showed that all eight samples fell below the 95% multivariate outlier threshold (χ^2^(2,0.95)=5.991, *p*<0.05)). The largest Mahalanobis distance was observed for S3 (D=2.125), followed by S8 (D=1.946); both remained below the predefined threshold (D=2.448). Thus, no sample was identified as a multivariate outlier, supporting the inclusion of all eight samples in subsequent analyses.**

5. **Global CpG methylations in sertraline- and saline-treated groups**

**Figure S12.** **Similar Global CpG methylations in sertraline- and saline-treated groups. Violin plots display the distribution of CpG methylation percentages for each group. A Mann–Whitney test was used, revealing no significant difference between the two groups (p > 0.05).**

**Figure S13. Cumulative distribution function (CDF) plot illustrating CpG methylation levels in heart tissue samples treated with saline or sertraline.** The y-axis represents the cumulative fraction of CpG sites with methylation levels at or below the corresponding value on the x-axis. Data were pooled from four saline-treated control samples (blue line) and four sertraline-treated samples (red line). A leftward shift of the sertraline curve compared to saline indicates global hypomethylation, whereas a rightward shift suggests hypermethylation in the sertraline group. These curves depict overall differences in DNA methylation patterns potentially caused by sertraline treatment. However, a Kolmogorov–Smirnov test showed no significant difference between the groups’ methylation distributions (D = 0.0206, *p* = 0.9269).

6. **Regional methylation levels of 23 regions**

**A**

**B**

**Figure S14. Regional DNA methylation profiles and differential methylation across the targeted genomic regions. (A) Volcano plot showing the relationship between regional methylation difference (ΔMeth) and statistical significance [−log10(*p*)] for all 23 targeted genomic regions. Each point represents one target region, with the two regions reaching statistical significance after Benjamini–Hochberg (BH) correction (*q* < 0.05) highlighted. (B) Heatmap showing Z-score-transformed methylation levels across the top 10 targeted genomic regions in eight samples (S1–S8). The 10 regions were selected based on ascending *q*-values and are labeled by gene ID and genomic coordinates. The color scale represents relative methylation levels, with red indicating higher and blue indicating lower relative methylation. The two FDR-significant regions, *Htr2a*_enhancer1 and *Tph2*_associated region, were among the top-ranked regions.**

7. **Sample information and sequencing quality**

**Table S2. Sample metadata**

| Sample ID | Treatment | Dam ID | Litter ID | Sex | Assay | Batch |
| --- | --- | --- | --- | --- | --- | --- |
| S1 | Saline | D1 | L1 | F | TBS | Batch 1 |
| S2 | Saline | D2 | L2 | M | TBS | Batch 1 |
| S3 | Saline | D3 | L3 | F | TBS | Batch 1 |
| S4 | Saline | D4 | L4 | M | TBS | Batch 1 |
| S5 | Sertraline | D5 | L5 | F | TBS | Batch 1 |
| S6 | Sertraline | D6 | L6 | M | TBS | Batch 1 |
| S7 | Sertraline | D7 | L7 | F | TBS | Batch 1 |
| S8 | Sertraline | D8 | L8 | M | TBS | Batch 1 |

**Table S3. Sample-level sequencing and alignment quality metrics**

| **Sample** | **Group** | **Unique alignment (%)** | **Mean target coverage (x)** |
| --- | --- | --- | --- |
| S1 | Saline | 75.0 | 176 |
| S2 | Saline | 75.1 | 147 |
| S3 | Saline | 74.6 | 204 |
| S4 | Saline | 75.1 | 175 |
| S5 | Sertraline | 77.5 | 173 |
| S6 | Sertraline | 76.6 | 144 |
| S7 | Sertraline | 77.4 | 202 |
| S8 | Sertraline | 77.4 | 173 |

8. **Site-specific Differential Methylation Analysis**

**Among the 1,411 captured on-target CpG sites, 619 showed an inter-sample methylation standard deviation (SD) >1%, and 414 showed an SD >2%.**

**Table S4. Site specific differential methylation (SD>1)**

| No. | Chr | Start | End | *p*-value | *q*-value | meth.diff |
| --- | --- | --- | --- | --- | --- | --- |
| 1 | 11 | 76997935 | 76997935 | 0.0023 | 0.331 | 2.71 |
| 2 | 11 | 76998124 | 76998124 | 0.0063 | 0.331 | -1.14 |
| 3 | 14 | 74639179 | 74639179 | 0.0023 | 0.331 | -10.8 |
| 4 | 14 | 74651767 | 74651767 | 0.0017 | 0.331 | 2.23 |
| 5 | 14 | 74732279 | 74732279 | 0.0678 | 0.331 | 0.86 |
| 6 | 17 | 81670202 | 81670202 | 0.0698 | 0.331 | 1.65 |
| 7 | 17 | 81670512 | 81670512 | 0.0712 | 0.331 | -1.05 |
| 8 | 7 | 82029890 | 82029890 | 0.0056 | 0.331 | 6.01 |
| 9 | 13 | 12106655 | 12106655 | 0.0033 | 0.331 | -0.85 |
| 10 | 13 | 12106758 | 12106758 | 0.0044 | 0.331 | -1.73 |

**Table S5. Site specific differential methylation (SD>2)**

| No. | Chr | Start | End | *p*-value | *q*-value | meth.diff |
| --- | --- | --- | --- | --- | --- | --- |
| 1 | 14 | 74639179 | 74639179 | 0.0023 | 0.679 | -10.8 |
| 2 | 14 | 31208378 | 31208378 | 0.0032 | 0.679 | 10.8 |
| 3 | 11 | 76998656 | 76998656 | 0.0650 | 0.774 | -1.49 |
| 4 | 11 | 76998840 | 76998840 | 0.0543 | 0.774 | 2.42 |
| 5 | 10 | 115175695 | 115175695 | 0.0163 | 0.774 | -8.92 |
| 6 | 10 | 115175830 | 115175830 | 0.0700 | 0.774 | -5.72 |
| 7 | 10 | 115076973 | 115076973 | 0.0415 | 0.774 | -7.04 |
| 8 | 14 | 74639632 | 74639632 | 0.0472 | 0.774 | -4.76 |
| 9 | 14 | 74639745 | 74639745 | 0.0306 | 0.774 | 16.8 |
| 10 | 14 | 74639877 | 74639877 | 0.0649 | 0.774 | -8.07 |
